# Possible Spillover of Pathogens between Bee Communities Foraging on the Same Floral Resource

**DOI:** 10.3390/insects12020122

**Published:** 2021-01-29

**Authors:** Anne Dalmon, Virgine Diévart, Maxime Thomasson, Romain Fouque, Bernard E. Vaissière, Laurent Guilbaud, Yves Le Conte, Mickaël Henry

**Affiliations:** INRAE—Unité Abeilles et Environnement—Site Agroparc—Domaine St Paul—228, Route de l’aérodrome CS40509, CEDEX 9, 84914 Avignon, France; virginie.dievart@inrae.fr (V.D.); maxime.thomasson@hotmail.fr (M.T.); romain.fouque13@orange.fr (R.F.); bernard.vaissiere@inrae.fr (B.E.V.); laurent.guilbaud@inrae.fr (L.G.); yves.le-conte@inrae.fr (Y.L.C.); mickael.henry@inrae.fr (M.H.)

**Keywords:** spill-over, virus, non-*Apis*, apoidea, hymenoptera, biodiversity, interspecific transmission

## Abstract

**Simple Summary:**

Floral resource availability is one of the keys to preserving the health of bee communities. However, flowers also present a risk of pathogen transmission, as infected pollinators could deposit pathogens while foraging, exposing other pollinators to infection via the consumption of contaminated nectar or pollen. Here, we studied, over time, the prevalence of seven viruses in bee communities that share the same small surface of floral resource in order to assess the risk of virus spillover. In total, 2057 bee specimens from 30 species were caught, identified and checked for the presence of viruses. Specimens from the Halictidae family were the dominant wild bees. The prevalence of viruses was quite high: at least one virus was detected in 78% of the samples, and co-infections were frequent. The genetic diversity of the viruses was also investigated to look for the possible association of geographic origin or host with shared ancestry.

**Abstract:**

Viruses are known to contribute to bee population decline. Possible spillover is suspected from the co-occurrence of viruses in wild bees and honey bees. In order to study the risk of virus transmission between wild and managed bee species sharing the same floral resource, we tried to maximize the possible cross-infections using *Phacelia tanacetifolia,* which is highly attractive to honey bees and a broad range of wild bee species. Virus prevalence was compared over two years in Southern France. A total of 1137 wild bees from 29 wild bee species (based on COI barcoding) and 920 honey bees (*Apis mellifera*) were checked for the seven most common honey bee RNA viruses. Halictid bees were the most abundant. Co-infections were frequent, and *Sacbrood virus* (SBV), *Black queen cell virus* (BQCV), *Acute bee paralysis virus* (ABPV) and *Israeli acute paralysis virus* (IAPV) were widespread in the hymenopteran pollinator community. Conversely, *Deformed wing virus* (DWV) was detected at low levels in wild bees, whereas it was highly prevalent in honey bees (78.3% of the samples). Both wild bee and honey bee virus isolates were sequenced to look for possible host-specificity or geographical structuring. ABPV phylogeny suggested a specific cluster for *Eucera* bees, while isolates of DWV from bumble bees (*Bombus* spp.) clustered together with honey bee isolates, suggesting a possible spillover.

## 1. Introduction

Animal pollination is essential for the reproduction of the majority of flowering crops and wild plant species [1]. Both the honey bee *Apis mellifera* and wild bees contribute to pollination [2,3]. Non-*Apis* insect pollinator species include wild bees, wasps, syrphid flies and butterflies [4]. However some species face a population decline [5,6] and experience a reduced abundance and a range of contractions, as described in the UK for bumblebees [7] in regards to total extinction. The decline of bumble bee populations is widespread in the northern hemisphere [8,9]. Additionally, this decline is not limited to *Bombus* spp. as other species of pollinators are also affected in terms of species diversity [10], abundance [11] or distribution [12]. 

Managed colonies of honey bees have also experienced severe losses since the end of the 1980s [6,13], but analysis of large sets of data have suggested a gradual replacement of wild bees by honey bees in the Mediterranean basin over the last 50 years [14]. Many different stressors have been shown to affect pollinator populations. First, the environment has been considerably modified by human activities. The increase of urban areas has led to habitat losses and fragmentation, leading to reduced wild bee species richness and functional diversity [15,16,17]. Intensification of agriculture has modified agricultural landscapes, and often reduced food availability for pollinators, and it requires the use of pesticides, which has been shown to impact both managed [18] and wild pollinators [19]. Commercial insecticides can impact even more wild bees than the honey bee, by reducing the nesting of solitary bees and their reproduction, and the colony growth of bumble bees [20]. 

Parasites and pathogens also contribute to pollinator decline. To date, more than seventy viruses have been detected in the honey bee alone (reviewed in Beaurepaire et al. [21]. From this impressive list, a lot of viruses have been described from high-throughput sequencing, and their epidemiology and functional significance for their host still needs to be elucidated. Seven of these viruses are known to induce measurable symptoms, such as the *Deformed wing virus* (DWV), which has been associated with colony losses [22,23,24,25,26,27]. This virus has three main variants, DWV-A, DWV-B and DWV-C with still debated respective virulence [28,29,30,31,32,33,34], and several recombinant variants have also been described [35,36,37,38,39,40]. *Black queen cell virus* (BQCV) and *Sacbrood virus* (SBV) can impair the developmental stages. The paralysis viruses cause trembling and paralysis of the bees. *Acute bee paralysis virus* is genetically close to *Kashmir bee virus* (KBV) and *Israeli acute paralysis virus* (IAPV), and these can be considered together as an “AKI-complex” group [41]. In addition to trembling and paralysis, *Chronic bee paralysis virus* (CBPV) can induce a darkening of the bees and greasy, shiny, and hairless bodies. Originally described in the honey bee, they have been shown to have a broader host range [42,43]. Symptoms of crumpled or aborted wings due to DWV have been observed in bumblebees and hornets and associated to virus replication [44,45,46,47], but this virus seems to be less prevalent in wild bees than in *A. mellifera.* DWV was detected in seven other wild bee genera, including bees from the Halictidae family [48,49], as reviewed in by [50,51]. Active replication for DWV was detected in the *Osmia* and *Melipona* bees [52,53]. Species from the AKI complex have been shown to replicate and impair individual fitness in several *Bombus* species [54], and were detected in five more other wild bee genera, including bees from the Halictidae family [50]. Conversely, CBPV does not replicate in pollinator hosts other than *A. mellifera*. Active replication of BQCV was also shown for *Bombus* spp. [55] and BQCV was detected in five other genera [48,50,56]. Furthermore, SBV was detected in four genera and the Halictidae family, but replication did not occur and its host range is supposed to be narrow [46,48,51]. Viral infections with viruses initially described in the honey bee are clearly not restricted to the honey bee host. In addition to pollinator hosts, CBPV, DWV, ABPV and KBV have also been detected in ants (Formicidae) and other arthropods. Evidence for their replication has been found in ants [57,58,59,60]. Further, experimental transmission of ABPV from the honey bee resulted in impaired ants locomotion and decreased emergence and colony size [59]. 

Even if already widespread, the current extent of the virus host range is probably underestimated, which is of major concern in assessing the risks of emerging diseases related to host switching. Symptoms are most often related to high virus titers, when persistent infections result in covert and symptomless infections. Commercial trade of asymptomatic honey bees and bumble bees have been shown to increase virus prevalence in sympatric wild species [47,61,62]. In addition, other solitary bees, such as mason bees that are used for commercial pollination (e.g., *Osmia cornuta*, *O. bicornis*) can be infected and multiply several viruses [47]; see Gisder et al. [51] for review). However, little is known about the role of other wild bees as virus reservoirs, and even more so if they could act as primary or secondary hosts. As virus genetic diversity is more structured by location than host species, the same virus variants that are shared within bee communities seem to circulate among pollinators [40].

Interspecies transmission is mostly expected to occur via oral-fecal transmission, given that the efficient *Varroa destructor* vector for the honey bee [63] does not affect other bees. CBPV [64], KBV [65], IAPV [48], DWV and BQCV [66] have been detected in honey bee faeces. While foraging, infected bees may contaminate the pollen, nectar, and flower surfaces, resulting in virus exposure for other pollinators. Indeed, flowers have been suggested to be hotspots for the horizontal transmission of some pollinator pathogens, including viruses [48,61,62]. SBV, BQCV and DWV have been detected in pollen pellets and further transmission to non-infected bees has been shown [48,52,67]. Moreover, consuming bees by carnivors may be another route of transmission. Indeed, several wasps have been detected as positive for many viruses, and active replication of DWV, AKI-complex and BQCV has been shown [45,68,69,70,71]. When infected, other horizontal routes of infection related to the social structure and life history traits of the wild bees (trophallaxis, larval food) may increase intra-specific transmission (see Yañez et al. [50] for review). 

This study aims to (i) characterize the virus community shared by managed honey bees and wild bees foraging on the same floral resource, and (ii) identify potential evidence for virus spillovers between wild bees and a managed bee species. We used standard community ecology analyses to provide a general description of the shared virus communities among bee species and other flower-visiting insects. The experimental design was intended to enhance the putative spillover of viruses between pollinators sharing the same small surface of floral resource, and we planted the attractive but not endemic *Phacelia tanacetifolia* to enhance pollinator visits close to apiaries of the domesticated *Apis mellifera*. To assess virus prevalence among bee species or functional groups, we detected common viruses originally described in honey bees (DWV, SBV, BQCV, CBPV, ABPV and the close IAPV and KBV) by multiplex PCR. In particular, under the assumption of a virus spillover among sympatric species foraging on the same resource, we would expect an increasing prevalence level in wild bees as time lapses since the onset of resource flowering. In addition, if viruses are transmitted between sympatric species, we would not expect host-specific clustering of the virus sequences from phylogenetic analyses.

## 2. Materials and Methods

### 2.1. Bee Sampling

The overall experiment was based on the assumption that a virus spillover might be identified by monitoring, over time, the shared virus communities in bee species and functional groups that foraged locally on the same mass-flowering resource.

We therefore created the ecological conditions necessary for the emergence of a local virus spillover by growing *Phacelia tanacetifolia*, an attractive flowering crop often used in pollinator-friendly agro-environmental schemes [72], in four 0.03 to 0.12 ha plots bordering the experimental apiaries (10 to 20 colonies at 2 to 10 m). While native of North America, this plant is fairly common in agro-environmental schemes in Europe, and is probably one of the most attractive plants for bees and other flower visiting insects [73]. It is also well suited for the rather hot and dry Mediterranean climate of the study region [74,75]. We were confident that the proximity of apiaries would attract large numbers of honey bee foragers, as well as a diverse wild bee fauna and other flower visiting insects, therefore increasing the chances of detecting potential virus spillovers over time. We net-sampled foraging bees in the plots using random walk transects [76] using a 2- to 4 person.h^−1^ sampling session per day, weather permitting (as soon as the temperature raised above 20 °C in the early morning), from the onset of flowering until its end. Along with each wild bee capture session, a sample of 40 foraging honey bees was captured and pooled for virus molecular analyses. Sampling began 5 to 8 days after the first flower was recorded (from Julian day 133 to 155) to allow for the first floral visits before sampling. Samples were collected at least twice a week throughout the blooming period (Supplementary Table S1), from Julian day 138 to 175.

On each sampling day, captured wild bees were maintained dormant at 4 °C, identified to genus whenever possible, and ultimately assigned to homogeneous morphotypes intended to become monospecific individual pools. Identification was performed under a binocular microscope according to bee morphological keys for France and Western Europe. The identification keys of local interest are reviewed and provided for each bee family by the French expert naturalist association Osmia (https://oabeilles.net/bibliographie/cles-de-determination). Individuals from the Halictidae family, belonging to *Lasioglosum* or *Halictus* genera, were pooled according to their size. Tubes were then stored at −80 °C. 

We varied pool sizes from 1 (a single individual) to 15 individuals, which was the largest size our sampling regime could afford on a daily basis. Species pool homogeneity was subsequently confirmed from molecular evidence. 

The first study year (2014) was dedicated primarily to set up a pilot study intended to validate the experimental design and to assess the most parsimonious individual pool sizes liable to reveal adequate virus prevalence in wild bees. As it appeared, on the one hand, unrealistic to obtain large monospecific pools for wild bees, and on the other hand, because we were unsure whether virus prevalence could be conveniently described using single individuals or few individuals per pool, we therefore favored an exploratory sampling strategy in a single pilot plot of phacelia (300 m^2^, 43°54’57, 81” N, 4°52’33, 50” E) close to an apiary, thereafter referred to as the Yr1 pilot plot. Virus composition in pools of sampled wild bees was affected by pool size (see Results), underlying the need to standardize pool size. During the second experimental year (2015), as a trade-off between experimental resolution and productivity, we pooled individuals by 10 whenever possible in a daily session, and further favored single-individual samples otherwise. This strategy ensured a broad virus prevalence screening in a species-rich bee assemblage (individual bee samples), and an in-depth virus prevalence focus on less common bee species (10-individuals pools).

We further included additional sampling plots to gain insights from spatial and temporal variations, aware of the variations that may occur over the season and across regions [26]. Here, we assessed whether infection levels in honey bees and wild bees remained consistent at short spatial and temporal scales, i.e., at distances shorter than most individuals’ foraging ranges and at timescales equivalent to a single blooming event. We again grew phacelia in the same pilot plot on the second year (Yr2 pilot plot), with the same apiary proximity, as well as a second nearby plot (1200 m^2^) 32 m away (Yr2 nearby plot), and a third 560 m^2^ plot, located 3.6 km away (43°56’48.61” N, 4°51’44.98” E), bordering another apiary (Yr2 distant plot).

### 2.2. Virus Detection

#### 2.2.1. RNA Extraction

A primary homogenate was prepared by grinding individuals pooled by morphotype (Supplementary Materials Table S1) into 500 to 800 µL phenol/guanidine-based QIAzol Lysis Reagent (Qiagen, Courtaboeuf, France) by homogeneization with one 8 mm diam. bead in 2 mL tubes, with tissue lyzer (Qiagen) during 30 s at 30 Hz. This was repeated three more times, after a wait of 30 s. Tubes were then centrifugated at 4 °C for 2 min at 12,000× *g*, and the supernatant was collected in a new tube to which chloroform was added (100 µL chloroform for 500 µL phenol/guanidine-based QIAzol Lysis Reagent (Qiagen), vortexed for 15 s and incubated at room temperature for 3 min. After 15 min centrifugation at 12,000× *g* at 4 °C, the aqueous phase was transferred for RNA purification on columns and treated according to the manufacturer’s protocol (RNAeasy minikit, Qiagen). The final suspension volume was 100 µL, and RNA was quantified on Nanodrop.

For *A. mellifera* samples, 40-individuals were pooled and RNA extracts were processed using standard methods, as described by Dalmon et al. [38]. Large specimens of *Xylocopa* and *Bombus* individual wild bees were ground in 5 mL PBS 1× using plastic bags containing filter. Five hundred microliters of this primary homogenate (filtered extract) was added to 900 µL of Quiazol (Qiagen, Courtaboeuf, France). After 5 min at room temperature, 180 µL of chloroform was added and samples were processed, as described above. 

#### 2.2.2. RT-PCR

Reverse transcription was performed from 1 µg RNA in a 20 µL total volume using random priming, according to the manufacturer’s protocol (High capacity RNA to cDNA, Life technologies^®^, Carlsbad, Californie, CA, USA). Ten microliters from the resulting cDNA were used to perform a multiplex PCR reaction with 2.5 mM MgCl_2_, 10 pmol of each primer, [77,78], 0.2 mM dNTPs, green buffer for GoTaq^®^ (Promega, Madison, WI, USA) and 1 unit of Go Taq G2 Flexi DNA polymerase (Promega, Madison, WI, USA), in a 25 µL total volume, using an Eppendorf Mastercycler^®^ (Eppendorf, Hambourg, Germany) Nexus SX 1 or MJresearch PTC100^®^ (BioRad, Hercules, Californie, CA, USA) thermocyclers. The following program was used: one cycle at 94 °C for 5 min, 35 cycles at 94 °C for 30 s, 56 °C for 30 s, and 72 °C for 45 s, and a final elongation cycle at 72 °C for 10 min. Amplified fragments code for part of the structural proteins, except for CBPV (partial RNA dependent RNA polymerase sequence). Two negative controls (for reverse transcription and PCR) were included for each run. One microliter of samples from 2014 was analyzed using Agilent High Sensitivity DNA chips (Life Technologies^®^) in 2100 Bioanalyzer (Agilent^®^) (Agilent Technologies, Santa Clara, Californie, CA, USA). Because the expected specific bands were easy to separate (expected specific bands for CBPV = 774 bp; KBV = 641 bp; BQCV = 536 bp; ABPV = 460 bp; SBV = 342 bp; DWV = 269 bp; IAPV = 158 bp), and did not require such a high sensitive electrophoregram, further PCR products (5 µL) were run in 4% agarose gel.

#### 2.2.3. Prevalence and Sequencing

In total, 237 positive samples from the multiplex assay were amplified in a single PCR performed with specific primers of each virus to be sent to Genoscreen for purification and Sanger sequencing. Four samples from a French collection were added to phylogenetic studies (*B.hortorum*-2011, *Bombus*2-DeuxSevres2012, *B.pratorum*-2012, and *O.aurulenta*-DeuxSevres2012). 3 µL of the 10-fold diluted cDNA were added to 0.5 µM of forward and reverse primers, 2.5 mM MgCl_2_, 0.2 mM dNTPs, green buffer for GoTaq^®^ and 1 unit of Go Taq G2 Flexi DNA polymerase (Promega, Madison, WI, USA), in a 25 µL total volume, and PCR conditions were the same as described above. Complementary sequencing was performed for DWV in the helicase coding region, using 5992f 5′-TCCTATTGCTGAATGTAGTC-3′ and 6693r 5′-GTTCACGACGCTTACTACAC-3′ [79] or in the untranslated region using ITR5 5′-CGATTTATGCCTTSCATGCG-3′ and LP3f 5′-TACGTTCTTGCTCCGCGCC-3′ [38]. The following program was used: one cycle at 94 °C for 2 min, 40 cycles at 94 °C for 30 s, 55 °C for 50 s, and 72 °C for 1 min, and a final elongation cycle at 72 °C for 10 min. Five microliters of the PCR products were run in 1.5% agarose gel to control the purity and band size. 

Sequences were checked for homologies in data banks using BLAST. Maximum likelihood phylogenies were built using MEGA version 6 with boostrap resampling (1000 iterations). Sequences were submitted to Genbank under the accession numbers MW435683 to MW435746 (SBV); MW442594 to MW442650 (BQCV); MW442651 to MW442713 (ABPV); MW442714 to MW442727 (5′end of DWV); MW442728 to MW442746 (DWV-Helicase).

### 2.3. Barcoding

To identify the species of the insect captured, 4 µL of the 10-fold diluted cDNA (about 20 ng) were added to 0.4 µM of forward and reverse primers LCO-1490 and HCO-2198 [80], 1.3 mM MgCl_2_, 0.2 mM dNTPs, green buffer for GoTaq^®^ and 1 unit of Go Taq G2 Flexi DNA polymerase (Promega), in a 10 µL total volume, and PCR was run with the following program: one cycle at 93 °C for 3 min, then 35 cycles at 95 °C for 30 s—51°C for 45 s—72 °C for 1 min and a final elongation at 72 °C for 8 min. Two microliters of the PCR products were run in 1.5% agarose gel to control the purity and band size, and then remaining PCR products were sent to Genoscreen for Sanger sequencing. Sequences were submitted to the BOLD database (http://www.boldsystems.org/index.php/IDS_OpenIdEngine) to obtain the species name. 

### 2.4. Data Analysis

All statistical analyses were performed with the R software for statistical computing (R Development Core Team, 2008). We first characterized the sampled bee communities. In a second step, we assessed the associated virus species assemblages using community ecology analytical approaches.

#### 2.4.1. Characterizing the Sampled Wild Bee Communities

We described the sampled bee communities in terms of species richness, composition and abundance across time and study plots. Species richness values were compared among plots using rarefied and extrapolated first-order Jackknife species richness estimator (*vegan* package [81]. Species composition was compared among plots with a Permanova (Permutational Multivariate Analysis of Variance) using Bray-Curtis dissimilarity matrices and 999 random permutations of samples (vegan package). Generalized Linear Mixt Models (GLMMs, *lme4* package, [82] were performed to document the temporal changes in the abundance of the main wild bee functional groups in sampling plots, namely bumble bees, halictid bees and the other more rare bees pooled together. The *Bombus* species group was restricted to four common bumblebee species in our study, all displaying social habits with a queen and division of labour among workers. The halictid species group is usually composed of diverse, often small-sized, generalist bees with either solitary or social habits. All the other more rare bee species were pooled together for the sake of simplicity in the analyses of abundance variations. Honey bee abundances were not formally recorded because they were systematically present at saturating levels, as a result of the proximity of experimental apiaries, and always much more abundant than all other flower-visiting insects.

The number of captured bees was analyzed as a function of plots, time (days since flowering) and across functional groups using a negative binomial family distribution with a log-link function, which is recommended for count data that are subject to over-dispersion such as in capture surveys [83]. Due to the numerous zeros in the capture dataset, the residual normality and homoscedasticity requirements where not satisfactorily met. We therefore performed a zero-inflated model (ZI-GLMM) to enable the correct fit of capture data. Finally, the sampling effort in each capture day (range = 2 to 4 persons.h-1) was accounted for as an offset term in the model. 

#### 2.4.2. Virus Prevalence in Honey Bees and Wild Bees

Variations of virus prevalence among bees sampled at different plots and times (days since the onset of flowering) were analyzed using an approach of multispecies distribution modeling (Brown et al., 2014), based on a multivariate GLM framework hereafter referred to as MGLM. Species distribution models are typically used to decipher how the distribution of a taxon (species) is driven by environmental variables. By handling multispecies datasets, MGLMs allow for the inclusion of many species simultaneously in a single multivariate model. It combines a species occurrence matrix (presence-absence in samples) and an environmental data matrix (environmental description of samples) and then uses permutation tests to identify potential species occurrence responses to the environment. Therefore, the species occurrence matrix is simply the virus presence table in bee samples, while the environmental data matrix is the descriptive table of those bee samples, i.e., the capture plot and day since the onset of flowering. Ultimately, virus occurrence predicted by MGLMs may be merely interpreted in terms of prevalence. 

Additionally, for wild bees, we included bee taxonomic assignations and functional traits such as bee sample characteristics liable to drive virus prevalence. Taxonomic variables were either the bee family, genus or species identity. Whenever a bee sample could not be assigned a species name based on COI molecular evidence, we kept the taxonomic resolution to the genus level. Functional trait variables were chosen based on their potential influence on virus prevalence, namely the level of sociality (solitary, gregarious or (eu-) social species) and the pollen diet specialisation (monolectic, oligolectic, or polylectic habits).

The potential associations between virus prevalence and bee sample characteristics were assessed in four steps, namely (i) a complete virus distribution model computation, (ii) a backward stepwise model simplification, (iii) a parsimonious model assessment and (iv) post-hoc univariate model confirmations.

*(i)*  *Complete virus distribution model computation*. We first fitted MGLMs for binomial presence-absence data with the traitglm function of the *mvabund* R package [84]. Complete models were computed, with all environmental variables, including: sampling plot identity and days since the onset of flowering, as well as taxonomic and functional assignations in the case of wild bee samples. The significance of the complete model was tested using a multivariate analysis of variance using 999 random permutations of samples among environmental variables.
*(ii)*  *Backward stepwise model simplification*. Whenever a significant environmental effect was detected, we further simplified the model by dropping non-significant environmental variables. We used the Akaike information criterion (AIC) to guide model simplification, considering a trade-off between model fit and complexity. Model simplification was iteratively pursued until only a subset of significant environmental variables was included. The resulting model was therefore viewed as the most parsimonious candidate model.
*(iii)*  *Parsimonious model assessment*. As a final model simplification step, we recomputed the most parsimonious model using the LASSO penalty function that automatically drops irrelevant virus × environment combinations explaining the overall variations of virus prevalence models. This algorithm permits further highlighting of the most salient virus prevalence patterns throughout the many possible combinations.
*(iv)*  *Post-hoc univariate model confirmations*. We are first and foremost interested in the potential rise of virus prevalence as time lapses since the onset of flowering. Whenever MGLMs detected such a temporal pattern, we performed posteriori univariate confirmatory analyses, focusing on each virus species of interest separately. To do so, we used a GLMM framework for binomial family date, specifying the number of days since the onset of flowering as a fixed variable, while controlling for the non-independency of samples proceeding from the same sampling plot and year using the plot identity as a random grouping variable.

## 3. Results

### 3.1. Characterising the Sampled Wild Bee Communities

A total of 2059 flower-visiting insects were captured and processed, among which 920 honey bees, 934 bees from genus Lasioglossum (Halictidae), 91 specimens of Bombus, 94 bees from 8 other genera or families, as well as 20 other non-apoid insects (syrphid flies, Polistes and Scolia wasps, Table 1). 

A total of 23 pooled samples of honey bees were analyzed (*n* = 40 individuals per sample) and 265 for the other insects (on average *n* = 4.3 individuals from the same morphotype per sample (range = [1–15], Table 1 and Supplementary Tables S1 and S2), 138 single individual samples and 49 10-individuals samples). A total of 183 out of the 265 wild bee samples could be successfully assigned to a species based on COI sequences (Table S2). The 72 other molecular samples were intractable due to insufficient ADNc quantities (*n* = 62), unsuccessful COI amplification (*n* = 5) or species mixtures in individual pools as a result of incorrect morphotype assignment (*n* = 5 samples only). 

**Figure 1:**
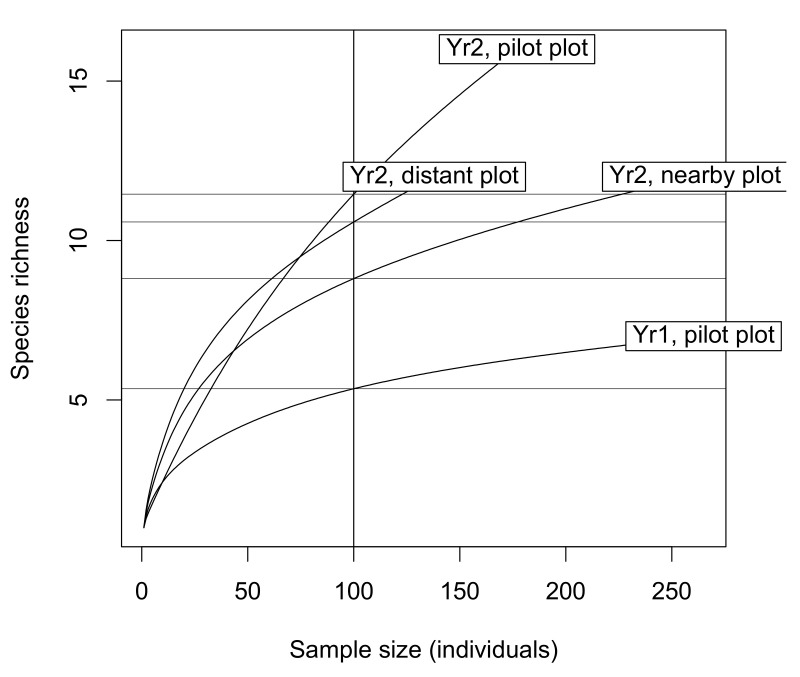
Wild bee cumulative species richness in the four study plots. Horizontal lines delineate the expected species richness rarefied to 100 sampled individuals in each study plot. See Table S3 for richness raw data and estimates.

In total, 29 distinct wild bee species were sampled in our study, with an expected total species richness S = 44.4 ± 5.1 (se) based on the first-order Jackknife richness estimator [85]. Both the observed and expected species richness varied substantially among the study plots (ranges [7–16] and [10.6–26.7], respectively). The largest richness contrast was associated with the inter-annual changes within the pilot plot (higher richness values during the second sampling year), while other plots harbored intermediate richness values (Figure 1). However, we found that even the contrasting richness estimates from the pilot plot had slightly overlapping 95% confidence intervals (Table S3), meaning that we cannot conclude to significantly different species richness among the four local communities (Yr1 and Yr 2 pilot plot, Yr2 nearby plot, Yr 2 distant plot).

The four local wild bee communities were largely dominated by *Lasioglossum malachurum*, a common social Halictid bee (*n* = 92 to 215 individuals per plot, total = 633 out of 1119 sampled wild bees, 56.6%). The second most sampled species (*n* = 45, found in rather balanced numbers among the plots) was *Bombus terrestris*.

Species composition was found to be rather homogeneous across time and space, as the Permanova (Table S4) returned no significant species dissimilarity among plots (*n* = 29 community samples out of 4 study plots, F = 1.67, *p* = 0.080), sampling dates since the onset of flowering (F = 1.35, *p* = 0.248), nor the interaction term (F = 1.041, *p* = 0.408). 

**Figure 2:**
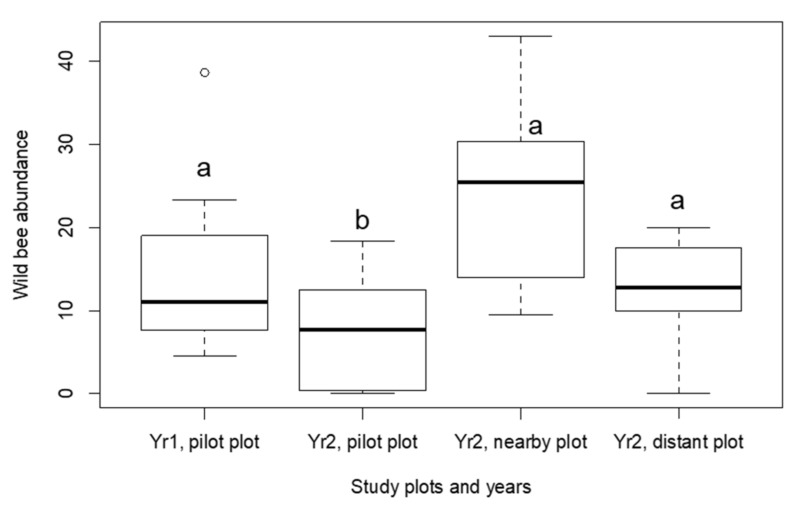
Wild bee abundance among plots. Different letters indicate significant differences between plots revealed by post-hoc pairwise Wald comparison tests (Table S5). (◦ represents classical boxplot).

In spite of the rather homogenous species composition, bee abundances significantly varied among plots and days of flowering, as revealed by the zero-inflated GLMMs. We first assessed abundance variations among plots, while specifying the three bee groups identities (bumblebees, halictid bees and other bees) as a random grouping variable. The overall wild bee abundance was significantly different among plots (Log-likelihood ratio test, *n* = 90 daily samples out of 4 study plots, Chi2 = 11.8, *p* = 0.008). Interestingly, the post-hoc pairwise comparisons revealed that these variations were mostly explained by the lower bee abundances in year 2 in the pilot plot (Figure 2), where the observed and expected species richness was the highest (Supplementary Figure S1). 

**Figure 3:**
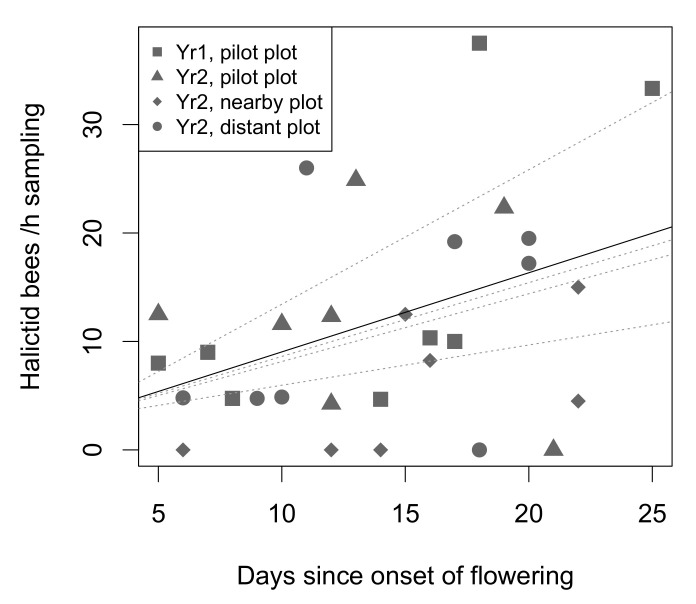
Halictid bee abundance increase over time. The solid line delineates the overall abundance temporal pattern, while dotted lines stand for individual plot patterns.

In a second step, we built a new GLMM, considering the plot identity as a random grouping variable to account for plot effects, and testing the effect of days of flowering, nested within wild bee functional groups. This allowed us to independently assess each bee groups abundance variations since the onset of flowering. Of the three tested groups, only halictid bees varied significantly, and positively, over time (*n* = 90 daily samples out of 4 plots, *z* = 3.501, *p* = 0.0004). The halictid bee abundance increase over time was consistent throughout the four plots (Figure 3).

### 3.2. Virus Prevalence in Honey Bees and Wild Bees

Viruses are widely distributed among honey bees and other pollinators: 79% of the pooled samples were positive for at least one virus, out of which 50.7% were positive for two viruses or more (Table S1). 

KBV remained undetected throughout the samples, both in the honey bees and in the other flower-visiting insects, but positive controls confirmed that our detection was valid. CBPV was only detected in the honey bees.

ABPV, BQCV, DWV and SBV were dominant in the honey bee samples (detected in 73.9% to 91.3% of the samples), while IAPV and CBPV were less common (13.0% and 17.4%, respectively, Table 1).

#### 3.2.1. SBV, BQCV, ABPV and IAPV Were Widespread in the Wild Bee Community

**Figure 4:**
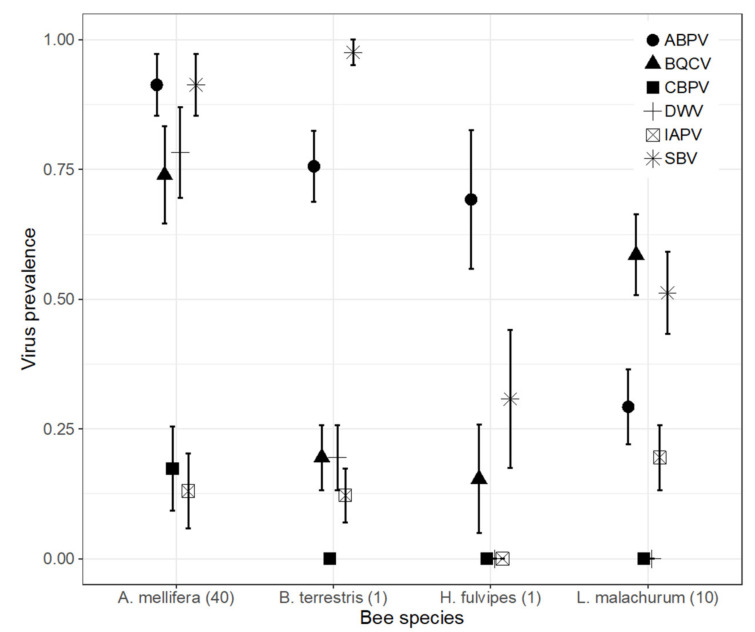
Descriptive plots of virus prevalence across samples of the most common species. Numbers in parentesis refer to the number of pooled individuals per sample. *A. mellifera*, *n* = 23 samples; *B. terrestris*, *n* = 43; *H. fulvipes*, *n* = 14; *L. malachurum*, *n* = 65 samples.

With the sole exception of CBPV, which remained undetected in all wild bee samples, SBV, BQCV, ABPV and IAPV were detected in the three main bee groups (halictid bees, *Bombus* spp. and other bee species)—though with various prevalence levels. SBV and BQCV were highly prevalent (>28%, up to 91.7% for *Bombus* spp. samples for SBV) in the most common species (Figure 2). ABPV was so highly prevalent in *A. mellifera* than DWV, and prevalence was also high in *Bombus* sp. and *Halictus* sp. (66.7% and 56%, Table 1), with prevalence in *Halictus fulvipes* higher than in other *Halictus* species (Figure 4). 

Highly prevalent in *A. mellifera* (78.3%), DWV was detected at low levels in *Bombus* sp, *Halictus fulvipes* and *Lasioglossum malachurum* (respectively in 13%, 8% and 1.3% of the samples, Figure 4).

Additionally, SBV, ABPV, BQCV and DWV were found in several of the 13 non-apoid insects (genera *Megascolia* and *Polistes*), except syrphid flies (Supplementary Tables S1 and S2). 

#### 3.2.2. Drivers of Virus Prevalence in Honey Bees

The virus distribution models computed with the multivariate GLM framework (MGLM) indicated that the overall virus prevalence in the honey bee samples was not dependent on the number of days since the onset of flowering, nor on the sampling plot (MGLM with 999 random permutations, *n* = 23 daily samples × 6 virus species, multivariate score = 15.88, df = 20, *p* = 0.578). The backward stepwise model simplification remained inconclusive for temporal trends in the six viruses (MGLM, multivariate score = 1.564, df = 5, *p* = 0.790) as well as for plot effects (multivariate score = 21.677, df = 18, *p* = 0.356).

#### 3.2.3. Drivers of Virus Prevalence in Wild Bees

Beside sampling days and plots, virus prevalence in wild bees could have been driven by social habits and dietary pollen specialization. We selected the single-individual samples for which COI species was confirmed and thus allowed us to obtain the complete functional information (sociality and diet specialization). This led to a subset of 103 samples belonging to 18 bee species from four families, with either solitary or social habits (respectively 10 and 8 species, Table S6). Social species use division of labor and cooperation to care for offspring, whereas in solitary species, females breed their own offspring. We also identified intermediate social habits, namely one gregarious species (*Andrena labialis*) whose individuals nest alone, but close to other conspecific nests, and one cleptoparasitic species (*Nomada distinguenda*) whose individuals are solitary but lay eggs in the nest of other bee species. Those were represented by a single individual each. For the sake of simplicity, we assigned them to the solitary category. Considering pollen diet specialization, a single individual was formally identified as belonging to an oligolectic species (*Andrena labialis*, specialized on the pollen of Fabaceae), while all other species are known to be polylectic. This unfortunately precluded any meaningful analysis of virus prevalence based on diet breadth. 

The wild bee virus distribution models (MGLM) were computed with all viruses, except CBPV, as it remained undetected in wild bee samples. We first performed the analysis on the 103 wild bee samples composed of a single individual. In a preliminary step, we computed complete models using either family, genus or species identity as candidate taxonomic drivers of virus prevalence. Family was by far the most parsimonious taxonomic resolution to account for virus prevalence, as it returned a much lower AIC value (ΔAIC >> 2) than the genus or species levels (AIC = 437.7, 450.8 and 488.0, respectively). 

**Figure 5:**
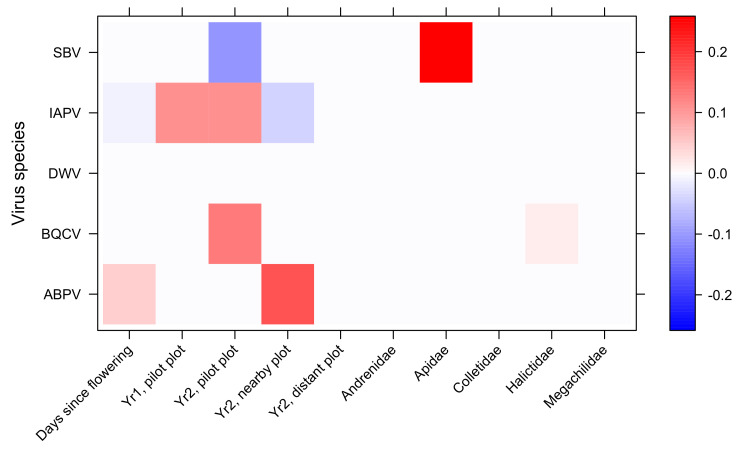
Summary of the most parsimonious virus distribution model (MGLM) in wild bees. Hot and cold colors stand for the standardized effect size (in standard-deviation units) of each quantitative driver or level of qualitative driver (horizontal axis) on the prevalence of each virus species (vertical axis). Grid combinations that do not contribute to increase the overall model likelihood are dropped (LASSO penalty function).

The complete virus distribution model returned an overall highly significant contribution of candidate drivers taken altogether (MGLM with 999 random permutations, *n* = 103 samples × 5 virus species, multivariate score = 126.3, df = 32, *p* = 0.004). However, the backward stepwise model simplification indicated that sociality was not a significant driver of virus occurrence. The most parsimonious virus distribution model included the plot effect, bee family, and days since flowering (Figure 5, Table S7). Most virus prevalence variations occurred among study plots, all other things being equal (Figure 5). Then, bee family effect was mostly supported by a strong positive occurrence of SBV in the Apidae family relative to other families. Finally, the days since flowering was positively related to ABPV prevalence, as one would expect from a spillover hypothesis, but this increase was not significant in the post-hoc model confirmation (Supplementary Figure S2). Conversely, IAPV prevalence decreased significantly over time, which goes against the spillover hypothesis (Supplementary Figure S2).

To further document the temporal trends of virus prevalence, which is likely to reveal a virus spillover as time lapses since the onset of flowering, we repeated the same analysis after selecting wild bee samples composed of 10 individuals. We focused on *Lasioglossum malachurum* samples only, which was by far the most frequently sampled wild bee species and the only one with a sample size large enough to support the analysis (*n* = 41 samples with 10 pooled individual). Those *L malachurum* samples only displayed IAPV, SBV, ABPV and BQCV viruses. Here again, virus distribution models revealed that virus prevalence was highly dependent on the sampling plot (MGLM, multivariate score = 52.32, df = 9, *p* = 0.001) and days since flowering (multivariate score = 29.15, df = 5, *p* = 0.001). The later temporal variations in virus prevalence were, however, not all significant, nor consistent with the temporal trends observed in single-individual bee samples (Supplementary Figure S2). 

### 3.3. Virus Phylogenies

**Figure 6:**
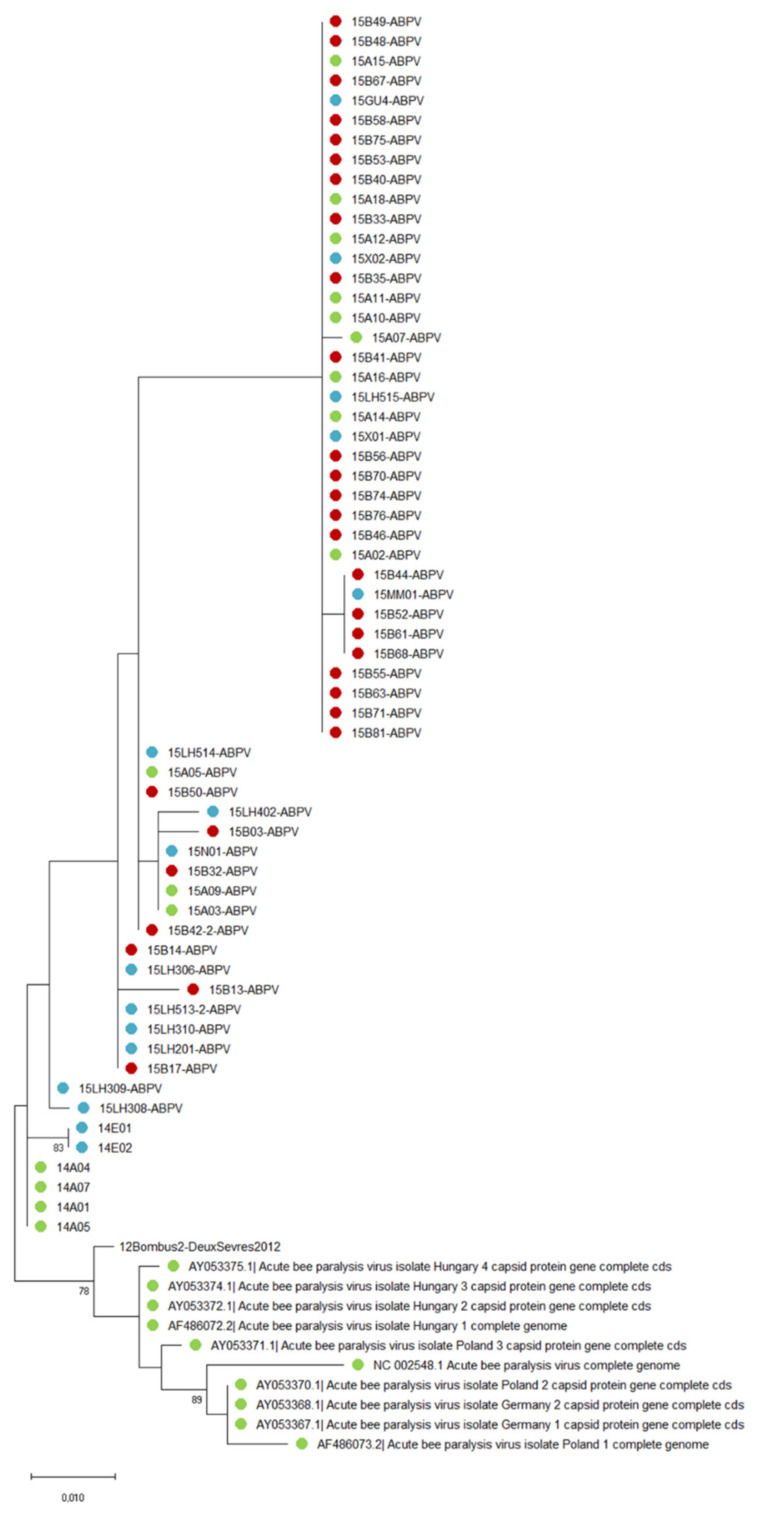
Maximum likelihood nucleotide phylogeny of ABPV sequences. ABPV sequences (*n* = 63) included the end of the intergenic region and the first 117 amino-acids of the capsid protein. There was a total of 421 nucleotides in the final dataset. The percentage of trees in which the associated taxa clustered together is shown next to the branches when >70% (nodes with >70% bootstrap support). Numbers correspond to the year of sampling, letters refer to genus and last numbers to sample code (listed in Supplementary Table S2). Sequences come from honey bees (● green circles); bumble bees (● red circles); other species (● blue circles).

A possible shared ancestry was investigated to look for a spatial and/or temporal structuring of the genetic diversity. Under the hypothesis of spillovers, virus isolates from different bee species are not expected to cluster separately as they share the same strains. All ABPV sequences from this study clustered in a large single clade (Figure 6), except the two sequences derived from *Eucera* (bootstrap = 83%), which notably included five more nucleotides in the intergenic region. Interestingly one *Bombus terrestris* sampled in 2012 in Northern France (12Bombus2-DeuxSevres 2012) was added to this study and clustered out of the Genbank sequences from other European countries, and out of the southern France sequence data from this study. Then, the French 2014–2015 strains from close locations are clearly distinct from the others in Genbank, which might suggest some geographic (and possibly temporal) structuring. 

IAPV sequences (*n* = 50 sequences) were too short (about 130 nucleotides) to produce a qualitative alignment and therefore no phylogeny could be built. However, similarities from BLAST clearly confirmed the assignation to IAPV species (data not shown). 

SBV sequences (310 nucleotides, *n* = 63 sequences) were close to the European SBV sequences available in databanks, but clustered out of the Korea/Thailand group (Supplementary Figure S3—Maximum likelihood nucleotide phylogeny of SBV capsid protein sequences). However, the clustering was not very strong (bootstrap support <70%), suggesting that this genomic region is not discriminant enough to observe some geographic structuring. 

From the 58 BQCV sequences (478 nt), no clear clustering could be observed (branches with bootstrap support <70%, Supplementary Figure S4), suggesting that this virus could be the same in all communities (*n* = 26 halictid bees, *n* = 11 *A. mellifera*, *n* = 10 bumblebees, *n* = 6 *Eucera*, *n* = 2 *Xylocopa*, *n* = 2 *Megascolia*, *n* = 1 *Osmia*), and/or that this genomic region may be very conserved. One *Bombus terrestris* sampled in 2012 in Northern France (12Bombus2-DeuxSevres 2012) was added to the set of data, but clustered with reference accessions. 

**Figure 7:**
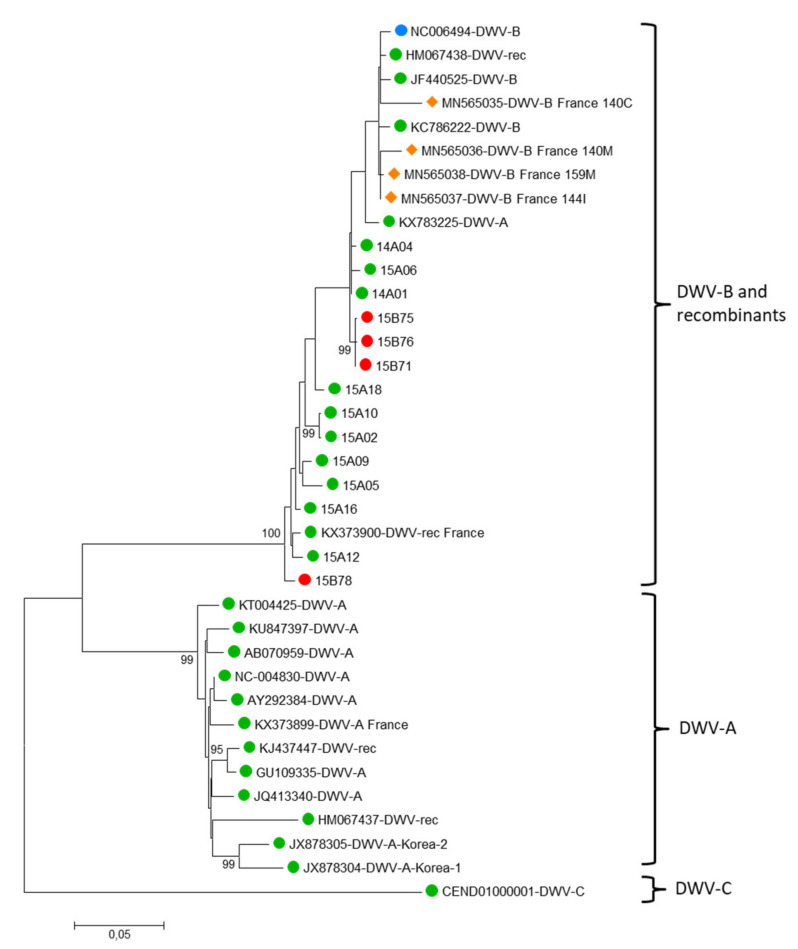
Maximum likelihood nucleotide phylogeny of DWV of the 5′ untranslated region and the region coding for the first aminoacids of Lp3. All ambiguous positions were removed for each sequence pair (pairwise deletion option). There were a total of 1214 positions in the final dataset, including 10 honey bee sequences and 4 bumble bee sequences from this study. Bootstrap values are shown for nodes with >70% bootstrap support. GenBank accession numbers and sample codes are associated with the DWV strain. Brackets show the main clusters corresponding to DWV strains. Numbers correspond to the year of sampling, letters refer to the genus, and the last numbers to sample code (listed in Supplementary Table S2). The sequences come from honey bees (● green circles); bumble bees (● red circles); ◆ hornets (orange symbol); *Varroa destructor* (● blue circles).

**Figure 8:**
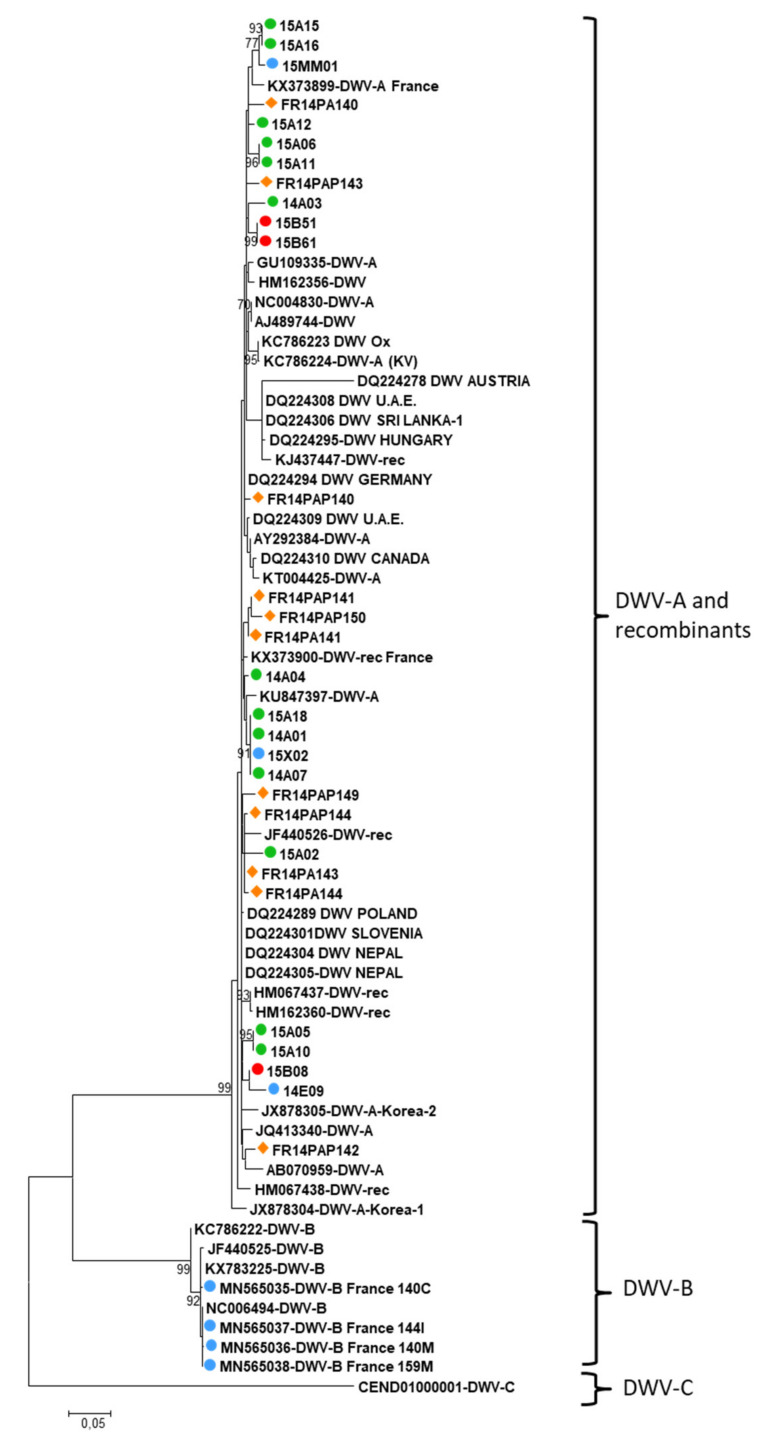
Maximum likelihood nucleotide phylogeny of DWV (sequences coding for the Helicase). All ambiguous positions were removed for each sequence pair (pairwise deletion option). There were a total of 638 positions in the final dataset, including 13 honey bee, 4 bumble bee, 11 hornet and 3 other species sequences from this study. Bootstrap values are shown for nodes with >70% bootstrap support. GenBank accession numbers and sample codes are associated with the DWV strain. Brackets show the main clusters corresponding to DWV strains. Numbers correspond to the year of sampling, letters refer to the genus, and the last numbers to sample code (listed in Supplementary Table S2). The sequences come from honey bees (● green circles); bumble bees (● red circles); hornets (◆ orange symbol); other species (● blue circles).

DWV was highly prevalent in honey bees, and was also detected to a lesser extent in bumble bees, and in few other wild bee samples (halictid bees, wasps, *Megascolia*, *Xylocopa* and *Eucera* samples, Table 1). Not all the samples detected as DWV-positive with the multiplex PCR could be amplified both in the untranslated region and in the region coding for the Helicase non structural protein, probably due to some variation in the sequence corresponding to the generic primers, or possibly to a mix of sequences for pooled halictid bee samples. Nevertheless, all sequences from this study corresponding to the 5′ end of the genome clustered with DWV-B and recombinant variants (Figure 7), while all sequences from the region coding for the Helicase clustered with DWV-A and recombinant variants (Figure 8). The 2014 and 2015 samples did not cluster separately, whatever the genome region under consideration. Nine sequences from honey bees were related to DWV-B in the 5′ UTR, and to DWV-A in the helicase coding region (14A01, 14A04, 15A02, 15A05, 15A06, 15A10, 15A12, 15A16, and 15A18, Figure 7 and Figure 8). For bumble bees, three sequences were related to DWV-A variants in the Helicase coding region (15B08, 15B51, 15B61, Figure 8), while four sequences clearly clustered with DWV-B and recombinants in the 5′ end of the genome (15B71, 15B75, 15B76, 15B78, Figure 7). Five clusters were derived from the Helicase coding region, one corresponding to two bumble bees and the other two including *Scolia flavifrons* (15MM01) and *Xylocopa iris* (15X02) with *A. mellifera* and *Eucera* (14E09) sequences, but unfortunately the 5′ end of the genome could not be amplified for these wild species, neither from the few DWV positive Halictid pooled samples.

## 4. Discussion

### 4.1. Patterns of Virus Prevalence in Bees and Other Flower-Visiting Insects

While describing the viruses detected in both honey bees and wild bees sharing the same floral resource, possible spillovers between species were investigated by way of virus prevalence and virus sequence shared ancestry. From the 29 wild bee species sampled in this study (1137 specimens), we identified 22 species as potential hosts for viruses originally described in the honey bee, and five of them belonged to genera in which viruses had never been detected before, to our knowledge (*Eucera*, *Megachile*, *Hylaeus*, *Nomada* and *Hoplitis*). The experimental design was set up to enhance and quantify processes of virus spillover among different groups of flower-visiting insects. Spillover, whereby a given virus is transmitted between two sympatric species or groups of species, is an example of indirect interference that can affect pollinators sharing the same foraging resources [86]. We expected spillover processes to occur locally in the course of the blooming phase of a highly attractive flowering crop exploited by an abundant and diversified pollinator fauna. Spillover might be evidenced by (i) an increase over the time of a virus prevalence in a given host species or group of species and (ii) by the absence of host-specific clustering of the virus sequences in phylogenetic trees. 

Indeed, we observed a high prevalence of the viruses originally described in the honey bee. From the whole sampling of pollinators (honey bees and other flower-visiting insects), 79% of the samples were positive for at least one virus, which is close to the prevalence already observed in other studies (80.4% in Dolezal et al. [87], Dolezal et al. [88]). Due to the pooled samples for the bees that were the most represented, this prevalence might be overestimated, because some individual bees may be sufficient to produce a positive signal for the whole pool of individuals. Piot et al. [89] have shown that sowing wildflower fields in landscape with few semi-natural elements could increase parasite (but not virus) prevalence in *Bombus pascuorum*, and was dependent on the size of the fields. In this study, the plots were much less than 1 ha in sub-urban areas, then our experimental design probably enhanced the pollinator presence in such attractive small plots and we could expect greater flower visitation rates. Flowers have been suggested to be parasite transmission hubs, because of the pathogens that infected bees may have deposited onto them [48,52,90], then increased visits may increase the risk for flowers to be contaminated. 

Apart from flower morphology, a range of other bee functional and morphological characteristics may contribute to explain virus transmissions through shared floral resources. Body size and tongue length set the propensity of bee species to get access to nectar rewards in tubular flowers with deep corollas. This theory predicts that long-tongued species will mechanistically be able to visit a broader range of flower morphologies than short-tongued species [91], thereby increasing the variety of transmission opportunities. Regardless of tongue length, small-bodied species may get easier access to deep corollas, on top of open corollas, again increasing potential transmission opportunities. However, small-bodied species also have shorter foraging flights than large species [92], which should reduce their spectrum of accessible flower diversity and increase virus transmission opportunities within their foraging range.

The close vicinity of the apiaries showing frequent co-infections (68% of the positive honey bee samples) could have also contributed to an increase in the presence of viruses on the flowers. As a consequence, virus prevalence from this study has to be considered in light of the experimental design that intended to maximize the risk for flowers to be visited by an infected bee, while typical prevalence in other areas would be expected to be lower. That could contribute to the high proportion of co-infections we observed (about 51% of the samples with at least 2 viruses), while McMahon et al. [93] reported less than 10% co-infections in *A. mellifera* and *Bombus* spp. Indeed, we observed a high prevalence of SBV and BQCV in wild bees (>28%), as already observed for SBV in other studies (45% in Dolezal et al. [88]; 38% in Levitt et al. [46]) when BQCV prevalence has shown more variability from one study to the other (predominant in hymenopterans [46] when only 3% in Dolezal et al. [88]). In addition, IAPV was detected more often in wild bees than in honey bees in our samples. This is congruent with previous observations where IAPV was highly prevalent in Andrenidae, and more prevalent in wild bees than in honey bees [88]. Levitt et al. [46] did not detect IAPV at all in honey bees when this virus occurred in seven of the orders of arthropods they collected. Such discrepancies in virus prevalence could suggest that wild bees are a reservoir for IAPV [48]. In addition the high ABPV and IAPV prevalence that we observed in wild bees is to be considered with the ability of ABPV to infect ant species, thereby reducing emergence and colony size, and impairing their locomotion and movement speed [59]. Ants were also supposed to be host for the close KBV [58]. Then, viruses from the AKI-complex are clearly not restricted to the honey bee. Viruses from the AKI-complex (KBV and IAPV) have been detected in the feces of the honey bee, and then the same could happen with wild bees. While foraging, bees may contaminate flower surface, the nectar and pollen with their feces that can be later consumed by other pollinators, although IAPV was not detected from pollen pellets of infected honey bees [48].

Viruses may also be transmitted through physical contacts among individuals. Indeed, CBPV has the ability to be transmitted by contact. However, even if present in the honey bee, it was never detected in wild bees. CBPV occurred only in 17.4% of the honey bee samples, then this prevalence may be too low for allowing a contact between infected honey bees with wild bees. Furthermore, if multiplex PCR detected CBPV, no typical symptoms of trembling, paralysis and darkening of the honey bees were ever observed, and the viral loads may have been below the expression threshold of 10^8^ virus particles per bee [94] and such a low virus load may not facilitate inter-individual virus transmission. 

Sociality was expected to foster virus prevalence. However, virus prevalence models did not identify social species as more prone to display high prevalence values. Instead, the taxonomic assignment of individuals was globally a better predictor of virus prevalence (Figure 5). From our sampling, DWV was more prevalent in social wild bee species, including *Bombus* spp. (less than 15% positive samples), and two social wasp *Polistes* spp., in accordance to previous findings in the UK [95] and congruent with DWV detection in several species of the *Vespula* and *Vespa* genera [45,46,48,95,96,97]. However, DWV remained undetected from the social halictid *Lasioglossum malachurum*, which was the most common wild bee species. Conversely, DWV may occur in carpenter bees *Xylocopa* spp. which are typically solitary—or with gregarious habits (the European *Xylocopa violacea* in the present study, Supplementary Table S1, and the South-American *X. augusti* in Reynaldi et al. [78]. DWV occurred at higher frequencies in other samplings from the USA (with the same prevalence in honey bees and wild bees of about 53% [88] and 66% including non—apoid species [46]). But different levels of prevalence are difficult to compare from one experiment to another, because they may be related to both the abundance and specific richness of the samplings, and also to the temporal dynamics of the infection. Alger et al. [98] never found DWV in bumblebees if foraging honey bees or apiaries were not present. By contrast, comparing the ubiquitous presence of DWV-C in *Melipona subtinida* to the much lower prevalence in the honey bee, de Souza et al. [53] suggested that *M. subtinida* may be a potential reservoir for DWV-C. 

### 4.2. Virus Prevalence Does not Provide Clear Insights on Possible Local Spillover Events

In our study, virus prevalence failed to reveal any virus occurrence pattern that was congruent with a spillover hypothesis. Globally speaking, the prevalence of the studied viruses in honey bees remained virtually unchanged throughout the flowering period and study plots, four of which being rather highly prevalent (ABPV, BQCV, DWV and SBV with an average prevalence >70% per 40-individual sample (Figure 4). Concomittently, none of the same viruses experienced significant prevalence increase over time in wild bees. Two of them (IAPV and BQCV) even significantly decreased in prevalence over time, in wild bees as a whole or in the abundant halictid bee *L. malachurum* (Supplementary Figure S2). Still, ABPV had a prevalence level displaying a marginally significant increase over time in wild bees, suggesting a possible spillover triggered by honey bees, but more samples would have been necessary to reach a definite conclusion.

We could find at least three reasons why quantifying prevalence patterns in pollinator species have hardly provided any evidence in support of a virus spillover hypothesis. First, pollinator species, and particularly bees in this study, consitute a very speciose fauna with a large suit of species often too rarely encoutered to allow appropriate sampling replication. Herein, only a handfull of the 29 wild bee species were sampled often enough to perform robust species-specific analyses (Figure 4). Pooling species into broad taxonomic or functional groups (Figure 4 and Figure 5) is certainly not optimal for detecting the spillover of virus species that are expected to be somehow host-specific. 

Second, the temporal variations of virus prevalence may be overwhelmed by the tremendous spatial variations. While bee communities were statistically similar among plots in terms of species composition, owing to shared dominent species, the assemblages of virus species differed greatly from one plot to another (Figure 5). Even the pilot plot and its nearby plot sampled during the same experimental year 2, which were only 30 m apart from each other, harboured substantial prevalence variations for most viral species.

Third, the abundance of flower-visiting insects may obviously vary during the flowering period, making it more difficult to interpret virus prevalence values. As a case in point, the halictid bee abundance increased 3- to 4-fold in study plots over the three-weeks of the *Phacelia* flowering sessions. This might be explained by a progressively increased attractiveness of the crop, or a concommitent emergence of newborn individuals. Such a large individual temporal turnover could possibly result in different hosted virus assemblages, and may contribute to explain the unexpectedly decreasing prevalence of IAPV and BQCV observed over time in wild bees, as a whole, and in *L. malachurum*, more specifically (Supplementary Figure S2). Despite an increased prevalence of parasites over time, both in honey bees and bumble bees, Graystock et al. [99] also observed that parasites decreased late in the season, while the floral abundance increased. 

### 4.3. Virus Phylogenies Provide Insigths on Possible Spillover Events

Phylogeny can provide information about a shared ancestry of the virus isolates from different species according to their geographical origin or original host species. Based on the lack of temporal or spatial structuring of the phylogenies, several authors have already suggested that viral strains may circulate freely between species, possibly because they may have similar virus receptors [46,48,93]. This study confirms that SBV and BQCV, although highly prevalent in various wild bees, do not show any host species structuring, nor spatial structuring, suggesting frequent spillovers between species. However, the BQCV sequence was highly conserved and possibly does not enable to detect small temporal or spatial variation. This may be corroborated by the existence of a separate cluster for ABPV from one *Bombus terrestris* sampled in 2012 in Northern France, while it did not cluster separately for BQCV. Nevertheless, we observed an interesting cluster for the new potential ABPV host *Eucera* spp bees. Unfortunately, we only have two ABPV sequences from the *Eucera* host, but they both show five nucleotides more in the intergenic region, which might be considered the hallmark of host-specific differentiations. Additionally, male aggregates described in this genus [100], by creating high contacts between males, may facilitate intra-species virus transmission.

Regarding DWV, the untranslated 5′ end of the genome was difficult to amplify from several samples, suggesting that primers did not anneal to the cDNA, probably due to mismatches caused by genetic variations, or possibly, for halictid bees, due to a mix of sequences in the pooled samples. However, when this region was amplified, we observed one cluster corresponding to bumble bee samples, close to DWV-B or French recombinant variants. In the more conserved Helicase coding region more samples could be amplified, and 5 clusters emerged within DWV-A and the French recombinant clade. No bumblebee samples could be amplified in both regions, and so we cannot conclude if some bumblebees were positive to DWV-A and others to DWV-B, or if they could have been recombinant variants exhibiting the 5′end of the genome as DWV-A and the region coding for the Helicase as DWV-B, and if one of these regions could not be amplified due to primer mismatching. However, the clustering of most of the bumble bee isolates with honey bee isolates in either of the two genomic regions with no strong evidence for population differentiation suggests that the same genotypes circulated in honey bees and bumble bees, and corroborates a recent spillover from honey bees to bumble bees, as described by Manley et al. [101]. Aside from the absence of bumble bee-specific structuring, we did not find evidence of a putative spatial or temporal structuring, in agreement with Singh et al. [48], but contrary to the strong temporal effect and spatial structure for DWV phylogeny shown by Levitt et al. [46] from 1217 nt of VP1. The recent evidence for bumble bee resistance to oral infection challenges the natural routes of infection from the honey bees to bumble bees [102]. Despite the fact that DWV replication was shown in bumble bee larvae and pupae artificially fed with the virus, natural infection of the larvae was not observed when they were fed by adult bumble bees continuously fed with high inputs of DWV. Additionally, these adults could not be infected as replication of the virus was not detected. Oral acquisition by adults, while foraging on contaminated flowers, still remains uncertain and suggests that other effective transmission routes may be involved for DWV spillover from honey bees to bumble bees. 

### 4.4. Deficient Knowledge on Virus Replication, Transmission, and Pathogenicity in Wild Bees

In this study, we detected the presence of viruses first described in the honey bee in 22 species never described as potential hosts before, but our experimental design does not indicate if it corresponds to a real infection, with the virus replicating in its host, or if the virus was just ingested with contaminated food. Replication was inferred from detection of the negative RNA strand for DWV in *Bombus* spp. [46,47,103,104], in eight other wild bees and wasps (*Vespula* spp., *Andrena haemorrhoa* [46,104]), *Vespa velutina* [97] and confirmed in controlled inoculation experiments in *Bombus* spp. [47]. All viruses from the AKI-complex replicate in *Bombus* [46,54,105,106], and negative replicative forms were also detected in *Vespa velutina* [68,107], as well as bees from the Halictidae family [46] and several ant species [58,59]. Finally, BQCV has been shown to replicate in *Bombus* spp. [55,104], and also in three wild bee species (*Anthophora plumipes*, *Xylocopa* spp., *Osmia bicornis* [104] and *Vespa velutina* [107]. Increasing descriptions of new hosts for viruses originally identified in honey bees raise the question of the reservoirs of these viruses, and the direction of the spillover. Prevalence data provide some information, but are not sufficient to conclude about the direction of the spillover. Transmission may follow a source-sink dynamics from the most infected species to the less infected ones, but the spillover may be bidirectional, and involve several successive hosts. The main reservoir could be a low prevalence host and involve a secondary host in the virus epidemiology, or other unknown non-sampled species may act as the main reservoir. 

Additionally, the pathogenicity of the viruses on the potential new hosts remains to be investigated. From our sampling, we did not observe any symptom, while wing deformations associated with DWV have been observed in bumble bees [44] and *Vespa velutina* [45], and a negative impact on individual host fitness has been suggested [47]. However when feeding bumblebee larvae or injecting adult bumble bees, Gusachenko et al. [102] could not reproduce any symptoms, so that the real impact of DWV in bumblebee communities still remains in question. Viruses from the “AKI” complex group (i.e., ABPV, KBV, IAPV) can reduce reproductive success in bumble bees and induce mortality [54,105], but no other effect has been described in wild bees. Experimental inoculation of two commercially-reared solitary bees *Megachile rotundata* and *Colletes inaequalis* by a mix lethal to honey bees (SBV 89.93%, IAPV 9.68%, DWV 1.2%, and BQCV 0.17%) did not impact their survival, and virus titers decreased over time, suggesting that these viruses were not able to be replicated in these species [88]. However, long-term effects on lifespan, reproduction, and overwintering success were not assessed. Investigating virus replication and virulence in the potential new hosts is now necessary to quantify the impact that these viruses may have on wild bees. In addition, the combination of stressors might also increase the impact of viral infections and have to be considered to understand the epidemiology of these viruses.

## 5. Conclusions

From the 29 wild bee species sampled, our study considerably extends the known host range of viruses originally described in the honey bee to five new genera and 22 species, detected positive for the first time, sometimes even from a few individuals. Sympatric species share viruses originally described in the honey bee, probably because flowers may act as transmission hubs. Host-switching may increase the risk to impact several wild pollinator communities. Directionality of the transfer is difficult to address from prevalence studies. However, based on both prevalence data and phylogenetic studies, we provide additional evidence that wild bees may act as reservoirs for some viruses, and that *A. mellifera* could disseminate DWV. Other viruses (SBV, BQCV) may be more ubiquitous than expected from their original description in the honey bees because host-specific clustering was never observed. With the increase of new virus descriptions using high-throughput sequencing, there is an urgent need to clarify the transmission dynamics of these putative pathogens, and to assess the risk they represent for pollinator communities. 

## Figures and Tables

**Table 1:** Number of positive samples per species (ranked in alphabetical order) for 7 viruses in honey bees and other flower visiting insects. Only samples representing at least 7 individuals are shown (complete data are available in Table S1). Prevalence (=percentage of positive samples) per genus (except for syrphi fliess, per family) is in bold. Thirty-two pooled samples of halictid morphotypes could not be assigned to a species.

Genus/Species	Number of Samples	Number of Pooled Individuals	ABPV	IAPV	KBV	BQCV	SBV	DWV	CBPV
***Apis mellifera***	**23**	**920**	**91.3%**	**13.0%**	**0.0%**	**73.9%**	**91.3%**	**78.3%**	**17.4%**
***Andrena*** **spp.**	**5**	**12**	**40.0%**	**20.0%**	**0.0%**	**20.0%**	**0.0%**	**0.0%**	**0.0%**
***Bombus*** **spp.**	**84**	**91**	**66.7%**	**19.0%**	**0.0%**	**28.6%**	**91.7%**	**13.1%**	**0.0%**
*Bombus terrestris*	43	45	31	7	0	9	41	8	0
*Bombus pascuorum*	7	7	5	1	0	1	7	0	0
*Bombus* sp.	29	34	17	8	0	12	24	2	0
***Eucera*** **spp.**	**9**	**55**	**22.2%**	**77.8%**	**0.0%**	**66.7%**	**55.6%**	**11.1%**	**0.0%**
***Halictus*** **spp.**	**25**	**56**	**56.0%**	**8.0%**	**0.0%**	**24.0%**	**28.0%**	**8.0%**	**0.0%**
*Halictus fulvipes*	14	17	10	0	0	2	5	0	0
*Halictus tectus*	4	15	0	0	0	2	1	0	0
*Halictidae* sp.	1	11	0	0	0	0	0	0	0
***Hylaeus*** **spp.**	**5**	**10**	**20.0%**	**40.0%**	**0.0%**	**0.0%**	**0.0%**	**0.0%**	**0.0%**
***Lasioglossum*** **spp.**	**77**	**676**	**26.0%**	**31.2%**	**0.0%**	**54.5%**	**41.6%**	**1.3%**	**0.0%**
*Lasioglossum malachurum*	65	633	17	22	0	35	28	0	0
*Lasioglossum pauperatum*	6	33	2	1	0	2	2	0	0
**undetermined *Halictidae***	**32**	**201**	**12.5%**	**9.4%**	**0.0%**	**21.9%**	**15.6%**	**0.0%**	**0.0%**
***Megachile*** **spp.**	**7**	**7**	**0.0%**	**0.0%**	**0.0%**	**0.0%**	**14.3%**	**0.0%**	**0.0%**
***Polistes*** **spp.**	**7**	**7**	**42.9%**	**0.0%**	**0.0%**	**28.6%**	**14.3%**	**28.6%**	**0.0%**
**Syrphidae spp.**	**2**	**9**	**0.0%**	**0.0%**	**0.0%**	**0.0%**	**0.0%**	**0.0%**	**0.0%**

## Data Availability

Sequences were submitted to Genbank under the accession numbers MW435683 to MW435746 (SBV); MW442594 to MW442650 (BQCV); MW442651 to MW442713 (ABPV); MW442714 to MW442727 (5’end of DWV); MW442728 to MW442746 (DWV-Hel).

## Supplementary Materials

The following are available online at https://www.mdpi.com/2075-4450/12/2/122/s1, Figure S1: Wild bee expected species richness among plots. Figure S2: Univariate post-hoc analysis of prevalence temporal trends. Figure S3: Maximum likelihood nucleotide phylogeny of SBV sequences corresponding to the capsid protein (n = 63). There was a total of 310 nucleotides in the final dataset. Numbers correspond to the year of sampling, letters refer to the genus and the last numbers to sample code (listed in supplementary table S2). Figure S4: Maximum likelihood nucleotide phylogeny of BQCV sequences corresponding to the capsid protein (*n* = 40). There was a total of 478 nucleotides in the final dataset. Numbers correspond to the year of sampling, letters refer to the genus, and the last numbers to sample code (listed in supplementary table S2). Tables S1 and S2: Complete description of the sampling and virus detection by multiplex PCR. Table S1: Number of positive samples per species for 7 viruses in honey bees and other flower visiting insects. The name of species detected positive for a virus for the first time is in red, genera are bolded and the sum of positive samples per genera indicated, except for honey bees where the species name is used as no other Apis species were sampled. Table S2: Sample characteristics (code, sampling date, plot, and duration (collection time (=CollectTime_h), number of collectors), number of pooled individuals in the sample, morphometry (body_Length), classification (family, genus, species, taxon, COI identification, percent identity), sociality, and level of lectism. Virus detection (ABPV, IAPV, KBV, SBV, BQCV, CBPV, DWV) is shown as detected = 1, or not detected = 0. Table S3: Descriptive results of observed and expected species richness in study plots, either estimated by the first-order Jackkniffe values, or rarefied to 100 individuals per study plot. Table S4: Results of the Permutational Multivariate Analysis of Variance (Permanova) comparing the species composition of sampled bee communities among sampling plots and dates. Table S5: Details of post-hoc pairwise Wald comparison tests contrasting wild bee abundance among sampling plots. Table S6: Taxonomic and functional description of the 18 wild bee species from single-individual samples used to study the drivers of virus prevalence. Table S7: Detailed results of the most parsimonious virus distribution model (MGLM) in wild bee samples composed of a single individual (*n* = 103 samples).
