# Supplementary material for: Possible Spillover of Pathogens between Bee Communities Foraging on the Same Floral Resource
Source: Insects. 2021 Jan 29;12(2):122. doi: 10.3390/insects12020122 (PMC7911050; doi:10.3390/insects12020122)
Supplement: Supplementary file 1 [file insects-12-00122-s001.zip › Figures S1–S4.docx]

**Figure S1.** Wild bee expected species richness among plots See Table S3 for richness raw data and estimates*.*

*
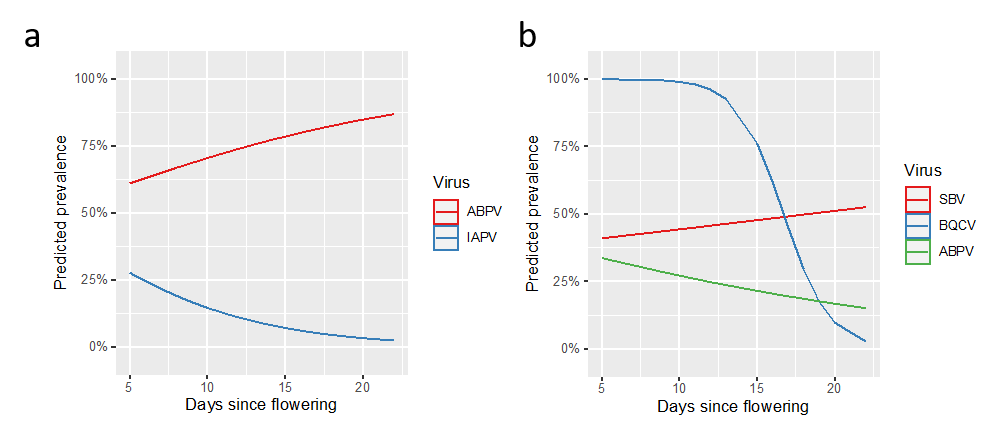
*

**Figure S2.** Univariate post-hoc analysis of temporal trends selected by the most parsimonious virus distribution models. (**a**) single-individual bee samples (n = 103): non-significant variation of ABPV prevalence (z = 1.707, *p* = 0.088) and a significantly decreasing IAPV prevalence (z = -2.364, *p* = 0.0181) ; (**b**) 10-individuals *L. malachurum* samples (n = 41): ): non-significant variation of SBV prevalence (z = -0.035, *p* = 0.972) and of ABPV prevalence (z = -1.301, p=0.193), but a significantly decreasing BQCV prevalence (z = -1.989, *p* = 0.047). * shows significant trends (*p* < 0.05).


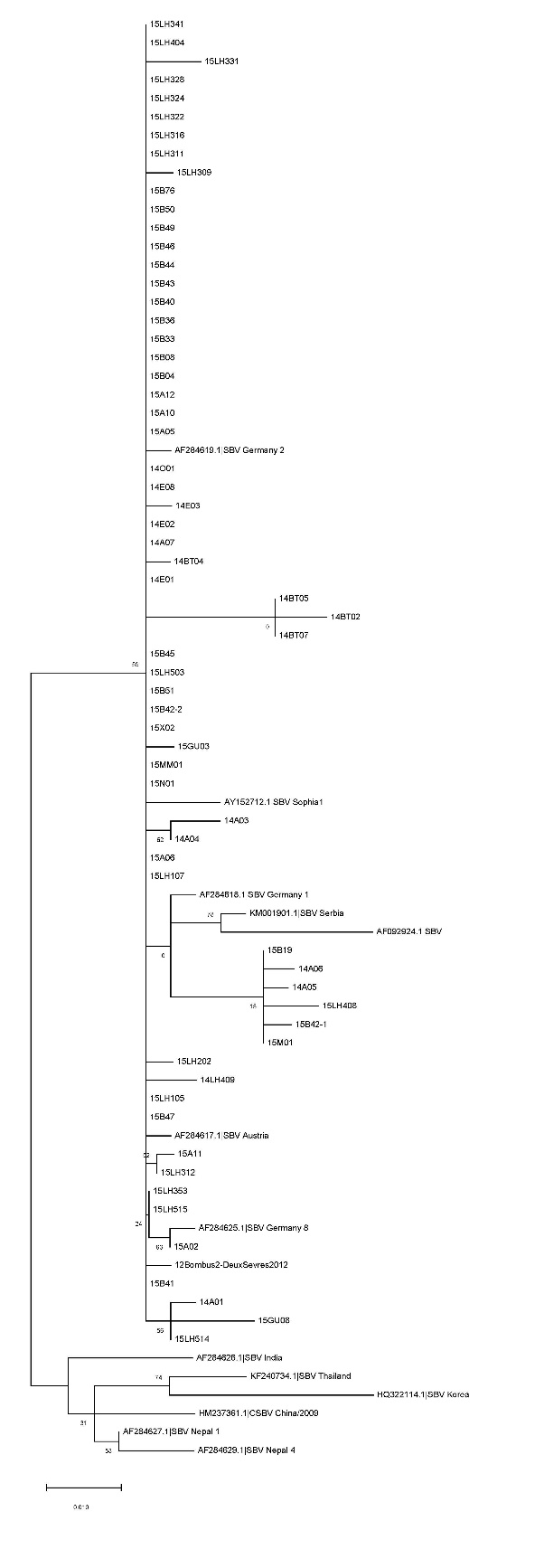


**Figure S3.** Maximum likelihood nucleotide phylogeny of SBV sequences corresponding to the capsid protein (n = 63). There was a total of 310 nucleotides in the final dataset. Numbers correspond to the year of sampling, letters refer to genus and last numbers to sample code (listed in supplementary Table S2).


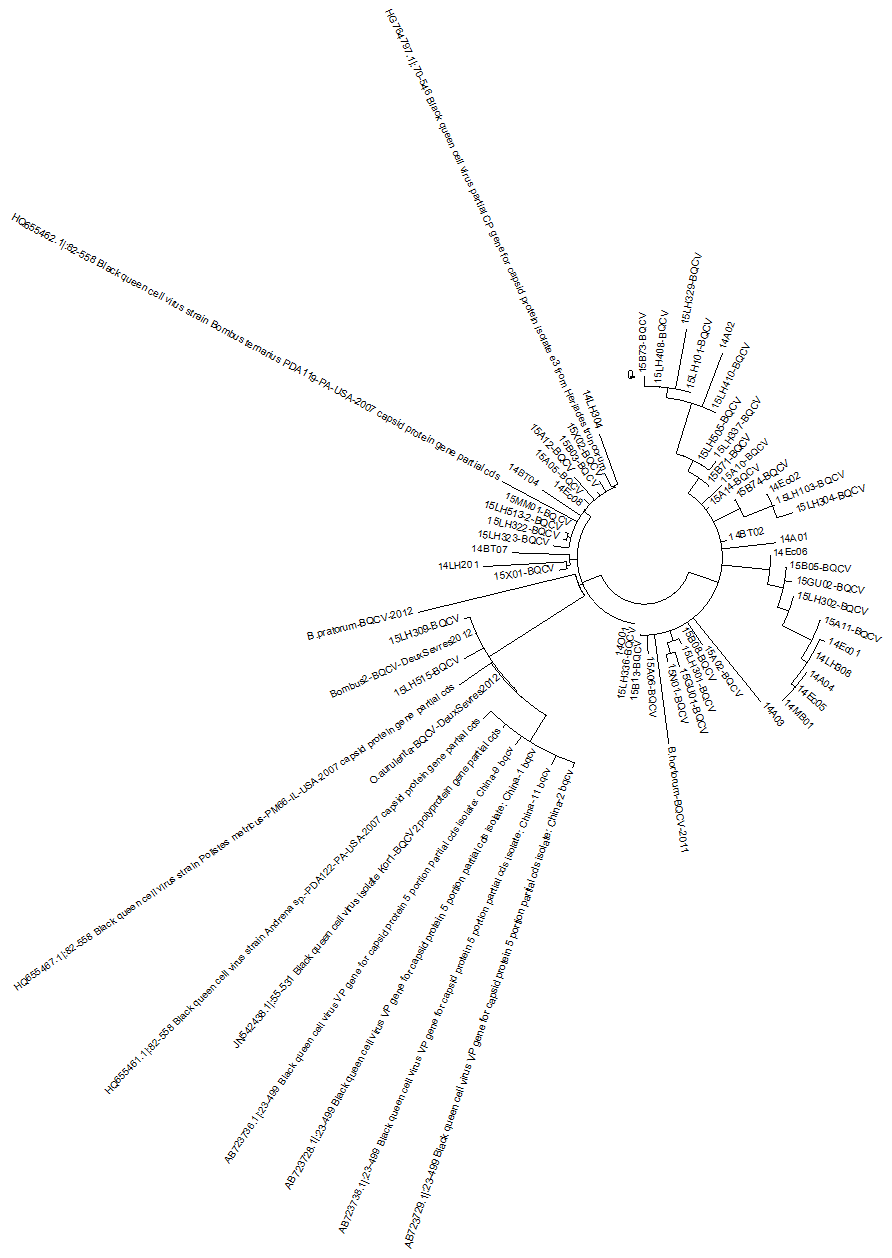


**Figure S4.** Maximum likelihood nucleotide phylogeny of BQCV sequences corresponding to the capsid protein (n = 66). There was a total of 450 nucleotides in the final dataset. Numbers correspond to the year of sampling, letters refer to genus and last numbers to sample code (listed in supplementary Table S2). All branches showed bootstrap values <<70% and sequences were >98% similar.
